# Exosome-Mimetic Nanovesicles from Hepatocytes promote hepatocyte proliferation *in vitro* and liver regeneration *in vivo*

**DOI:** 10.1038/s41598-018-20505-y

**Published:** 2018-02-06

**Authors:** Jun-Yi Wu, An-Lai Ji, Zhong-xia Wang, Guang-Hui Qiang, Zhen Qu, Jun-Hua Wu, Chun-Ping Jiang

**Affiliations:** 1 Department of Hepatobiliary Surgery, Affiliated Drum Tower Hospital of Nanjing University Medical School, 210008 Nanjing, Jiangsu Province China; 2 Department of General Surgery, The Affiliated Hospital of Yangzhou University, Yangzhou University, 225000 Yangzhou, Jiangsu Province China; 3 Department of Hepatobiliary Surgery, Drum Tower Clinical College of Nanjing Medical University, 210008 Nanjing, Jiangsu Province China; 4 Jiangsu Key Laboratory of Molecular Medicine, Medical School, Nanjing University, 210093 Nanjing, Jiangsu Province China

## Abstract

The liver has great regenerative capacity after functional mass loss caused by injury or disease. Many studies have shown that primary hepatocyte-derived exosomes, which can deliver biological information between cells, promote the regenerative process of the liver. However, the yield of exosomes is very limited. Recent studies have demonstrated that exosome-mimetic nanovesicles (NVs) can be prepared from cells with almost 100 times the production yield compared with exosomes. Thus, this study investigated the therapeutic capacity of exosome-mimetic NVs from primary hepatocytes in liver regeneration. Exosome-mimetic NVs were prepared by serial extrusions of cells through polycarbonate membranes, and the yield of these NVs was more than 100 times that of exosomes. The data indicated that the NVs could promote hepatocyte proliferation and liver regeneration by significantly enhancing the content of sphingosine kinase 2 in recipient cells. To the best of our knowledge, this is the first time that exosome-mimetic NVs from primary hepatocytes have been prepared, and these NVs have components similar to exosomes from primary hepatocytes and, in some respects, biofunctions similar to exosomes. Strategies inspired by this study may lead to substitution of exosomes with exosome-mimetic NVs for biofunctional purposes, including utilization in tissue repair and regeneration.

## Introduction

The liver is the most important organ for metabolism in the body and has strong tissue repair and regeneration potential. A variety of injuries and diseases can cause the liver to compensate by repair and regeneration when functional liver mass loss occurs. Liver regeneration is an extremely complex process that is subject to sophisticated regulation^1^. Studies of liver regeneration have very important clinical significance, for example, they assess the safety of liver transplantation to improve the survival rate and provide assistance to patients after surgical resection of pathological liver tissue. In recent years, many researchers have focused on the mechanisms of this process, in which growth factors and cytokines as well as numerous hormones are involved^2^.

To promote liver regeneration, several strategies have been explored, such as using trypsin inhibitors or augmenters of liver regeneration (ALR) and the use of subcutaneous liver tissue engineering^3–5^. In addition, certain types of nanoparticle carriers have been used as a new tool for the treatment of liver diseases^6,7^. Changing the expression of key genes and transcription factors, such as osteopontin (OPN), TGF beta and PP2Acα^8–10^, can also increase hepatocyte proliferation and liver regeneration. However, changing the expression of key genes and transcription factors may also cause many problems, such as tumorigenesis and insertional mutagenesis^11^. Only a few problems have been validated in the clinical setting^2^, so efficient and safe methods for promoting liver regeneration are urgently needed.

Recent studies have indicated that exosomes play an important role in promoting liver regeneration^12^. Exosomes are nanosized (30–100 nm) extracellular vesicles that can be secreted by a variety of cells, including hepatocytes, as communicators in the microenvironment, and play a diverse role in the regulation of cell activities through the transfer of biologically active proteins, lipids, and RNAs^13,14^. In addition, many studies have focused on the roles of exosomes in immunology and cancer biology^15,16^. Accumulating evidence has shown that exosomes can promote cell proliferation and angiogenic programs, which can accelerate the repair and regeneration of tissue^17–20^. With regard to the role of exosomes in the liver, the therapeutic capacity of exosomes for liver regeneration has been explored in recent studies because they are highly biocompatible and highly efficient. Tan CY *et al*. found that exosomes from mesenchymal stem cells could promote hepatic regeneration in drug-induced liver injury models through higher expression of proliferation-associated proteins^21^. In addition, exosomes from immature dendritic cells could induce tolerance in a rat liver allograft model^22^. Exosomes could also be developed as therapeutics for systemic insulin resistance and autoimmune liver diseases^23,24^. However, one of the obstacles for the application of exosomes in clinic is that the yield of exosomes from donor cells is very limited^25^. Thus, it is important to develop new methods to engineer exosomes^26,27^. To solve this problem, exosome-mimetic nanovesicles (NVs) with more than a 100-fold greater yield than exosomes have been shown to deliver information to recipient cells, and these NVs have certain properties that are similar to natural exosomes^28–30^. Furthermore, the use of exosome-mimetic NVs derived from pancreatic β-cells have been shown to cause bone marrow (BM) cells to be efficiently differentiated into therapeutic insulin-producing cells, which confirms that exosome-mimetic NVs have biological functions that are similar in some ways to natural exosomes^31^.

Hiroyuki Nojima *et al*. found that exosomes derived from hepatocytes could effectively promote hepatocyte proliferation *in vitro*^32,33^, and induce liver regeneration *in vivo* by transferring sphingosine kinase 2 (Sphk2) to recipient cells^33^. Because exosomes from primary hepatocytes can promote hepatocyte proliferation and liver regeneration and exosome-mimetic NVs contain molecules similar to those found in natural exosomes from primary hepatocytes, we hypothesize that exosome-mimetic NVs, derived from primary hepatocytes via multiple extrusions of cells through filters, may possess abilities similar to those of exosomes from primary hepatocytes and could thus function as intercellular communicators in the processes of liver regeneration. Therefore, in the present study, we prepared exosome-mimetic NVs from primary hepatocytes and investigated the therapeutic capacity of exosome-mimetic NVs from primary hepatocytes in liver regeneration and the associated mechanisms.

## Results

### Characterization of exosome-mimetic NVs

**Figure 1:**
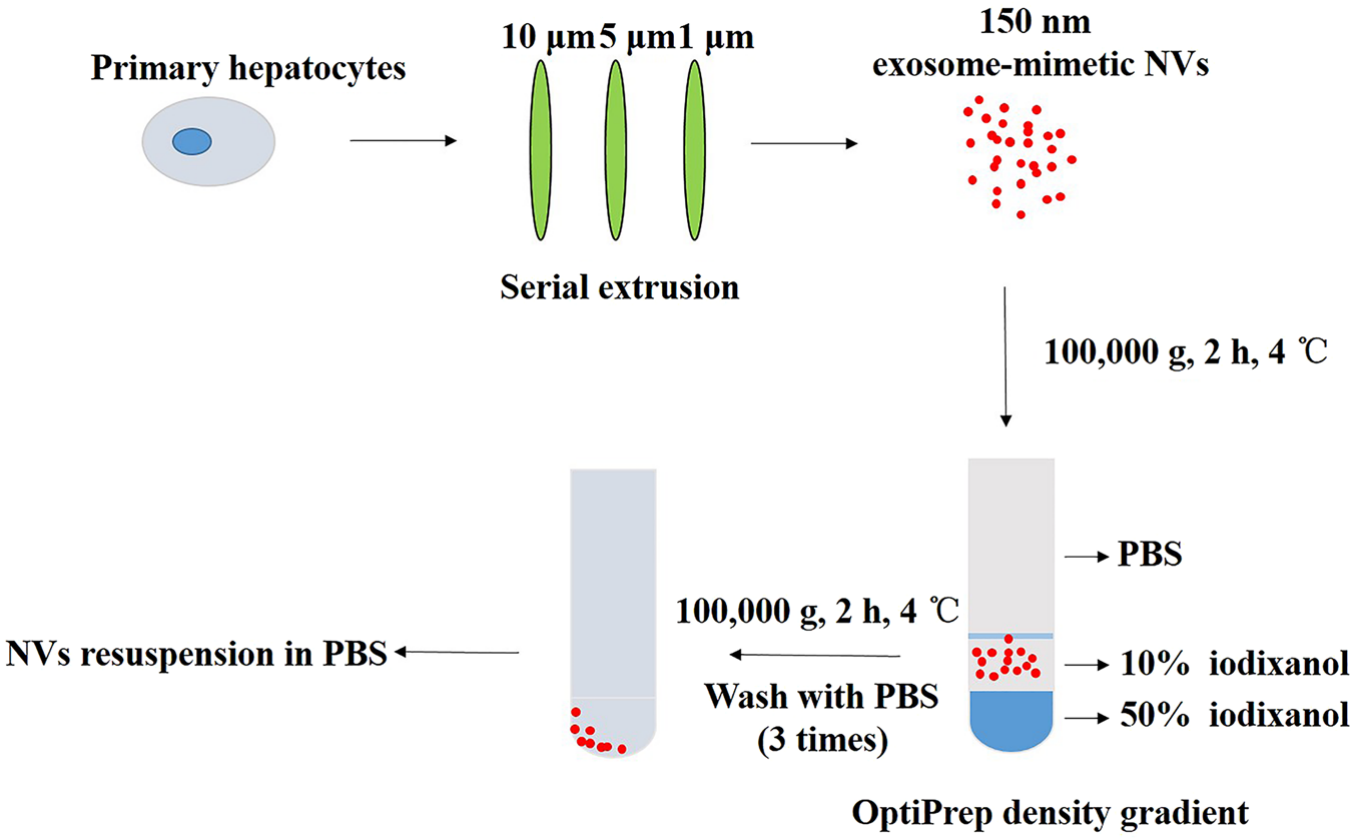
Schematic diagram of the preparation of exosome-mimetic NVs from primary hepatocytes.

**Figure 2:**
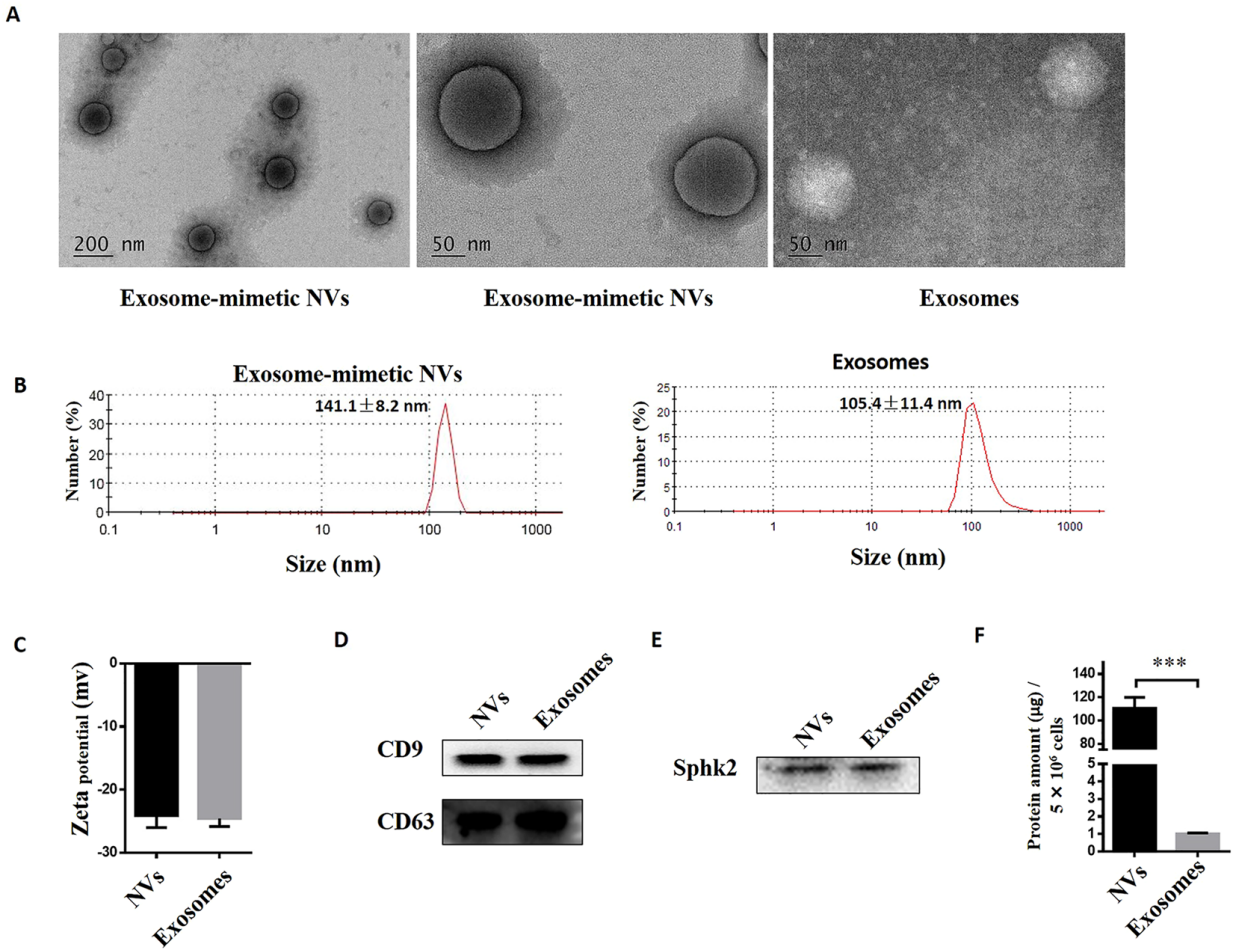
Characteristics of exosome-mimetic NVs produced from primary hepatocytes. (**A**) Representative TEM images of exosome-mimetic NVs. Scale bars: 200 and 50 nm. (**B**) (**C**) Size and zeta potential distribution of exosome-mimetic NVs and exosomes determined by a Zetasizer Nano ZS90 instrument. (**D**) Exosomal markers (CD9, CD63) on exosome-mimetic NVs were measured with western blotting. (**E**) Sphk2, which plays an important role in liver regeneration, in exosome-mimetic NVs and exosomes from hepatocytes was measured with western blotting. (**F**) The yield of exosome-mimetic NVs and exosomes from the same number of primary hepatocytes (5 × 10^6^ cells) was evaluated based on total protein in exosome-mimetic NVs and exosomes using a BCA protein assay. ***P < 0.001. Uncropped western blots are shown in Supplementary Figure 1.

The procedure for the preparation of exosome-mimetic NVs from primary hepatocytes is illustrated in Fig. 1. First, the NVs were prepared by serial extrusions through polycarbonate membranes with pore sizes of 10 μm, 5 μm, and finally 1 μm. Then, we used a two-step OptiPrep gradient, consisting of 10% and 50% iodixanol, to acquire purified NVs. An examination of the NVs by TEM revealed that the NVs retained a vesicular structure similar to exosomes (Fig. 2A). The size distribution of the NVs and of exosomes was measured using a Malvern Zetasizer, and the results revealed mean vesicle diameters of 141.2 nm and 105.4 nm (Fig. 2B), respectively, which were similar to the mean diameters measured with TEM (Fig. 2A). In addition, the zeta potential of the NVs and the exosomes was nearly the same (Fig. 2C). Additionally, we used western blotting to characterize the NVs and exosomes, and the results showed that the NVs contained common exosomal marker proteins, such as CD9 and CD63 (Fig. 2D). To characterize the molecular composition of the NVs with respect to hepatocyte-derived natural exosomes, we detected the proteins contained in the NVs and exosomes through mass spectrometry for the proteomic analysis. We identified 428 proteins contained in both the NVs and exosomes, which are listed in Supplementary Table S1. The most important aspect of exosome-mimetic NVs from primary hepatocytes was that they also contained Sphk2 (Fig. 2E), which is present in exosomes from primary hepatocytes and plays an important role in liver regeneration^33^. Furthermore, the NVs were produced with a more than 100-fold greater yield compared to exosomes (Fig. 2F), as measured by total protein content. All of these analyses showed that the NVs were similar to exosomes in several important ways, including diameter, size and specific protein characteristics.

### Exosome-mimetic NVs could induce hepatocyte proliferation ***in vitro***

**Figure 3:**
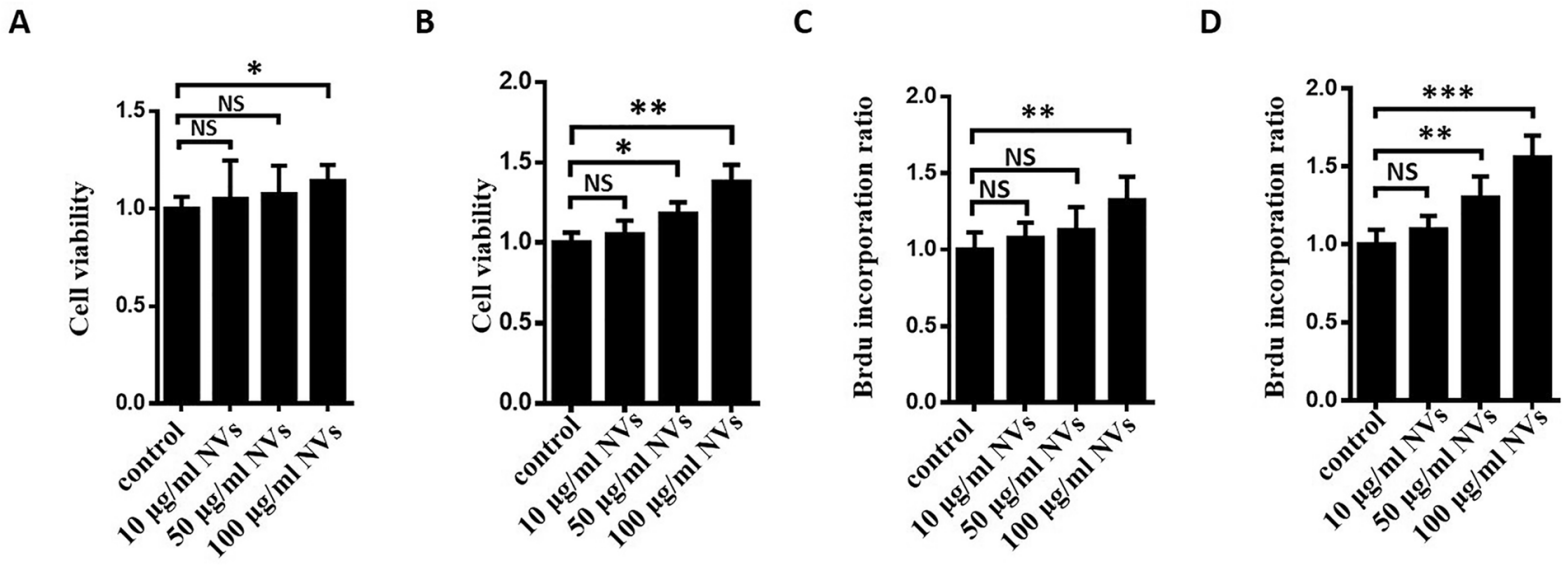
Exosome-mimetic NVs derived from primary hepatocytes induce hepatocyte proliferation *in vitro*. (**A**) Primary hepatocytes were treated with different concentrations of exosome-mimetic NVs for 24 h, and cell viability was measured using MTT assays. (**B**) Primary hepatocytes were treated with different concentrations of exosome-mimetic NVs for 48 h, and cell viability was measured with MTT assays. (**C**) Primary hepatocyte proliferation after treatment with different concentrations of exosome-mimetic NVs for 24 h was measured by BrdU incorporation. (**D**) Primary hepatocyte proliferation after treatment with different concentrations of exosome-mimetic NVs for 48 h was measured by BrdU incorporation. NS, not significant; *P < 0.05; **P < 0.01.

To investigate the roles of NVs in the proliferation of primary hepatocytes, we determined cell viability and BrdU incorporation after adding different concentrations of exosome-mimetic NVs to primary hepatocyte cell culture for different times. We found that treatment with NVs derived from primary hepatocytes for 24 h at 100 μg/ml significantly promoted hepatocyte proliferation (Fig. 3A), and the addition of exosome-mimetic NVs at 50 μg/ml for 48 h increased hepatocyte proliferation significantly (Fig. 3B). A dose-dependent increase in hepatocyte proliferation after treatment with exosome-mimetic NVs for 24 h or 48 h was also confirmed by BrdU incorporation (Fig. 3C,D). These results confirmed that exosome-mimetic NVs from primary hepatocytes have a biofunction similar to that of exosomes from primary hepatocytes.

### Exosome-mimetic NVs promoted liver regeneration ***in vivo***

**Figure 4:**
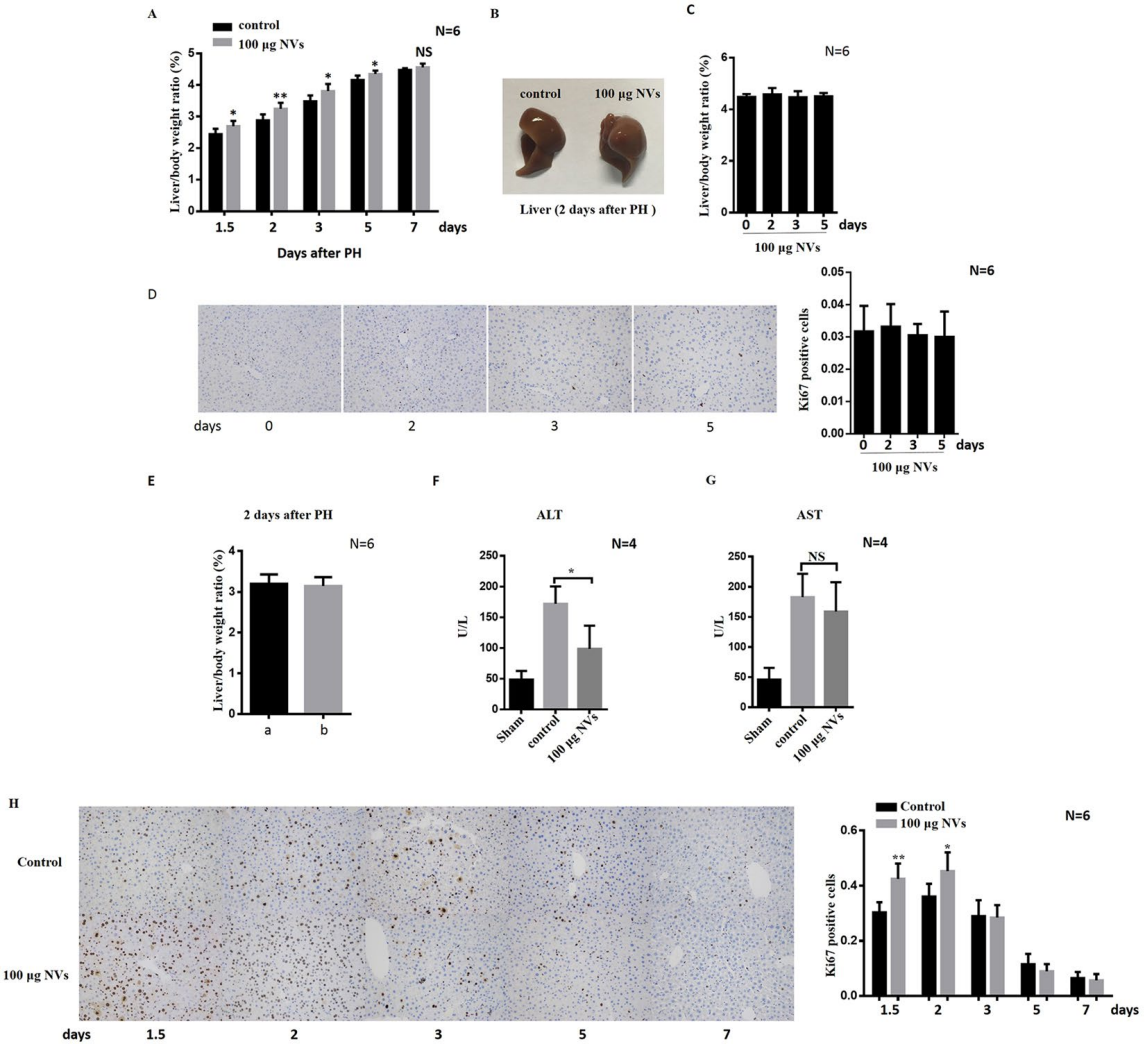
The effect of exosome-mimetic NVs on liver regeneration *in vivo*. (**A**) Immediately and every 24 h after hepatectomy, mice were treated with PBS (control) or 100 μg of exosome-mimetic NVs through intravenous injection, and liver regeneration was determined by calculating the liver-to-body weight ratio. (**B**) Representative livers from mice treated with PBS vehicle or exosome-mimetic NVs after PH, indicating the difference in liver size. (**C**) The effect of exosome-mimetic NVs in mice without partial hepatectomy. The mice were treated with 100 μg of exosome-mimetic NVs via intravenous injection every 24 h. (**D**) Typical images of Ki67 staining of livers from mice without partial hepatectomy. The mice were treated with 100 μg of exosome-mimetic NVs via intravenous injection every 24 h. (**E**) Liver-to-body weight ratio 2 days after PH. a, treatment with exosome-mimetic NVs isolated from mouse livers 2 days after PH. b, treatment with exosome-mimetic NVs isolated from normal mouse livers. (**F**,**G**) Serum AST and ALT levels in mice treated with PBS vehicle or exosome-mimetic NVs after PH. (**H**) Typical images of Ki67 staining of livers from mice treated with PBS vehicle or exosome-mimetic NVs at different time points. NS not significant; *P < 0.05; **P < 0.01.

To evaluate whether the exosome-mimetic NVs had any effect on hepatocyte proliferation and liver regeneration *in vivo*, we used a two-thirds PH mouse model, and then the NVs or PBS (as vehicle control) was injected intravenously immediately and every 24 h after PH. The liver mass was measured at different time points after PH, and serum ALT and AST and hepatocyte proliferation were measured 2 days after PH to investigate the effect of the NVs on liver regeneration *in vivo*. The data showed that compared with control group, the ratio of liver/body weight in the NV group increased significantly after PH. However, no significant difference was observed in absolute regeneration between the control condition and treatment with exosome-mimetic NVs 7 days after PH, which confirmed that the effect of exosome-mimetic NVs is persistent at early time points after PH (Fig. 4A). Two days after PH, the livers in the NV group were larger than those in the control group, which confirmed that treatment with exosome-mimetic NVs could promote liver regeneration (Fig. 4B). In addition, we evaluated the effect of exosome-mimetic nanovesicles in mice without partial hepatectomy. Because no liver injury is present in mice without partial hepatectomy, hepatocytes were in the G0 phase, in which mitotic division is rare or absent. We found that exosome-mimetic NVs could not increase hepatocyte proliferation *in vivo* without partial hepatectomy (Fig. 4C). In addition, the number of Ki67-positive cells in the mice without partial hepatectomy was not increased (Fig. 4D). We also found that NVs isolated from mouse livers 2 days after PH have effects similar to those of NVs isolated from normal mouse livers (Fig. 4E). Serum ALT and AST, which reflect the severity of liver injury and the functional status of the liver, were increased immediately after surgery, and were measured again 2 days after the PH. The serum activity of ALT recovered to nearly normal levels more quickly in the mice treated with the NVs than that in the control mice (Fig. 4F). However, the serum activity of AST was not different between the mice treated with NVs and the control mice treated with PBS (Fig. 4G). Furthermore, we found that *in vivo* hepatocyte proliferation, assayed by Ki67 staining, was significantly increased after treatment with 100 μg of NVs for 36 h and 48 h (Fig. 4H). The number of Ki67-positive cells in the mice represents the level of cell proliferation. These results suggested that NVs could effectively accelerate hepatocyte proliferation and liver recovery after PH and significantly promote liver regeneration *in vivo*.

### Exosome-mimetic NVs could be taken up by primary hepatocytes

**Figure 5:**
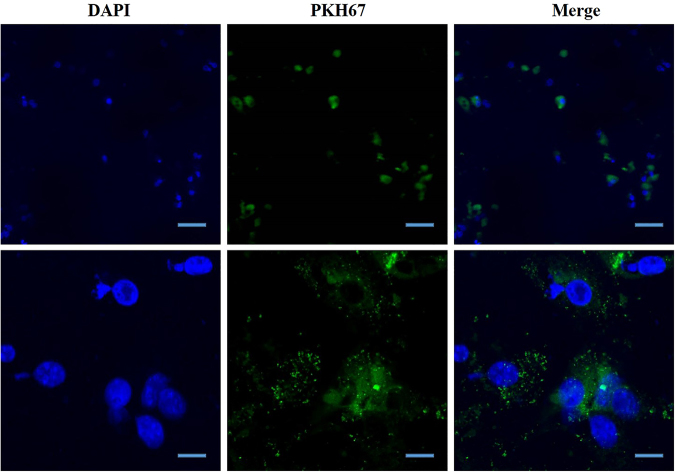
Uptake of primary hepatocyte-derived exosome-mimetic NVs by primary hepatocytes. Exosome-mimetic NVs labeled with PKH67 (green) were added to the culture of primary hepatocytes for 24 h. DAPI was used to stain the nuclei (blue). The cells were measured with fluorescence microscopy as described in Materials and Methods. Low-magnification images (Scale bars: 200 μm) and high-magnification images (Scale bars: 20 μm) of hepatocytes show that exosome-mimetic NVs were internalized into primary hepatocytes.

To verify whether exosome-mimetic NVs from primary hepatocytes could be taken up by primary hepatocytes, NVs labeled with PHK67, a cell membrane marker, were added to primary hepatocytes. After 24 h, the uptake of NVs by primary hepatocytes was detected with confocal microscopy. The results showed that NVs labeled with PKH67 could be absorbed by primary hepatocytes in a manner similar to exosome uptake (Fig. 5).

### Exosome-mimetic NVs can deliver sphingosine kinase 2 to recipient hepatocytes

**Figure 6:**
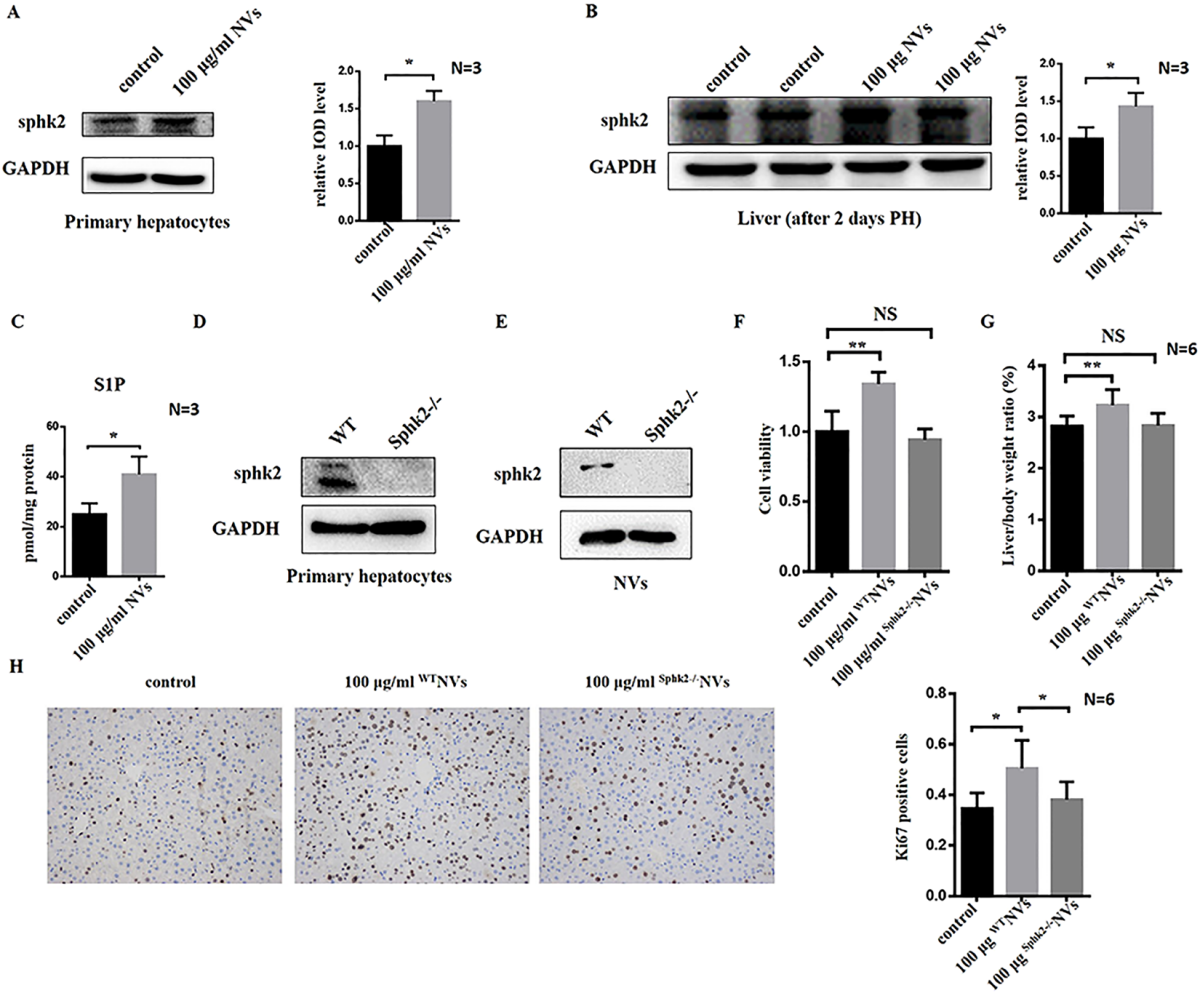
Exosome-mimetic NVs delivered Sphk2 to recipient cells *in vitro* and *in vivo*. (**A**) Primary hepatocytes from normal mouse livers were treated with exosome-mimetic NVs at a final concentration of 100 μg/ml for 48 h (NV group), and the level of Sphk2 in primary hepatocytes was determined with western blotting; PBS was used as a vehicle control (control group). The relative IOD level was also analyzed. (**B**) Immediately and 24 h after PH, mice were treated with PBS vehicle (control) or 100 μg of exosome-mimetic NVs from primary hepatocytes (NV group), and 2 days later, the levels of Sphk2 in livers were determined by western blotting. The relative IOD level was also analyzed. (**C**) Primary hepatocytes from normal mouse livers were treated with PBS (vehicle control) or exosome-mimetic NVs from primary hepatocytes at a final concentration of 100 μg/ml for 48 h, and the intracellular levels of S1P in primary hepatocytes were measured with an ELISA. (**D**) Sphk2 levels in primary hepatocytes from wild-type mice or SphK2−/− mice were measured by western blotting. (**E**) Sphk2 levels in exosome-mimetic NVs from wild-type mice- or Sphk2−/− mice-derived primary hepatocytes were measured by western blotting. (**F**) Primary hepatocytes from normal mouse livers were treated with exosome-mimetic NVs from wild-type mice- or Sphk2−/− mice-derived primary hepatocytes at a final concentration of 100 μg/ml for 48 h, and cell proliferation was assessed based on cell viability using MTT assays. control: PBS as vehicle control group; 100 μg/ml NVs: exosome-mimetic NVs from wild-type mice-derived primary hepatocytes at a final concentration of 100 μg/ml group; 100 μg/ml ^Sphk2−/−^NVs: exosome-mimetic NVs from Sphk2−/− mice-derived primary hepatocytes at a final concentration of 100 μg/ml group. (**G**) Immediately and 24 h after PH, mice were treated with 100 μg of exosome-mimetic NVs from wild-type mice- or Sphk2−/− mice-derived primary hepatocytes, and 2 days later, liver regeneration was determined by calculating liver-to-body weight ratio. Control: PBS as vehicle control group; 100 μg/ml NVs: 100 μg of exosome-mimetic NVs from wild-type mice-derived primary hepatocytes; 100 μg/ml ^Sphk2−/−^NVs: 100 μg of exosome-mimetic NVs from Sphk2−/− mice-derived primary hepatocytes. (**H**) Typical images of Ki67 staining of livers from mice treated with PBS vehicle, exosome-mimetic NVs from wild-type mice or exosome-mimetic NVs from Sphk2−/− mice. NS not significant; *P < 0.05; **P < 0.01. Uncropped western blots are shown in Supplementary Figure 1.

Hepatocyte exosomes have been confirmed to promote hepatocyte proliferation and liver regeneration by delivering Sphk2, which is abundant in exosomes from primary hepatocytes, to recipient cells^33^. SphK2 is an SphK isoform that produces S1P in the nucleus^34^. Because exosome-mimetic NVs from primary hepatocytes also contained Sphk2 (Fig. 2E), and in order to determine whether exosome-mimetic NVs from primary hepatocytes could deliver and increase the Sphk2 content in the recipient cells or the liver, we first examined the level of Sphk2 in hepatocytes and in liver that had been treated with exosome-mimetic NVs from primary hepatocytes. The data showed that treatment with 100 μg/ml NVs for 48 h increased Sphk2 levels in recipient primary hepatocytes from normal mouse livers (Fig. 6A). Additionally, treatment with NVs also enhanced Sphk2 levels in the liver 2 days after PH (Fig. 6B). This dramatic upregulation of Sphk2 confirmed that exosome-mimetic NVs from primary hepatocytes could promote hepatocyte proliferation *in vitro* and liver regeneration after PH *in vivo* by delivering Sphk2 to recipient cells and to the liver, increasing the level of Sphk2 in recipient cells and the liver.

Many studies have identified that the SphK isoform is responsible for the formation and release of S1P, and the production of S1P by SphK2 has been implicated as a significant regulator of cell proliferation^33,35^. To verify whether delivery of Sphk2 by exosome-mimetic NVs from primary hepatocytes could increase S1P levels in recipient cells, similar to exosomes from primary hepatocytes, we measured the levels of S1P in recipient cells via an ELISA. The data showed that intracellular levels of S1P in recipient primary hepatocytes from normal mouse livers were elevated significantly after treatment with 100 μg/ml NVs (Fig. 6C). All of these data confirmed that exosome-mimetic NVs from primary hepatocytes can function in a manner similar to exosomes derived from primary hepatocytes, which deliver Sphk2 to recipient cells to generate intracellular S1P, resulting in cell proliferation.

To further investigate the role of Sphk2 in hepatocyte proliferation, we compared the effect of exosome-mimetic NVs derived from primary hepatocytes from wild-type (abbreviated as ^WT^NVs or NVs) and Sphk2-knockout (^Sphk2−/−^NVs) mice on promoting hepatocyte proliferation and liver regeneration to confirm whether Sphk2 was a key cargo in the process. The data showed that primary hepatocytes from Sphk2−/− mice did not contain Sphk2 (Fig. 6D), and the NVs (^Sphk2−/−^NVs) from Sphk2−/− mice-derived primary hepatocytes also did not contain Sphk2 (Fig. 6E). Furthermore, the results showed that the promotive effect on hepatocyte proliferation and liver regeneration disappeared when we used ^Sphk2−/−^NVs instead of ^WT^NVs *in vivo* and *in vitro* (Fig. 6F,G). In addition, the number of Ki67-positive cells in the liver after treatment with ^Sphk2−/−^NVs instead of ^WT^NVs *in vivo* was significantly decreased (Fig. 6H). These results suggested that Sphk2 delivery by exosome-mimetic NVs to recipient cells and the liver could improve the level of intracellular S1P, which regulated the proliferation of hepatocytes and liver regeneration.

### Upregulation of SphK2/S1P by exosome-mimetic NVs activated AKT and ERK phosphorylation in hepatocytes and livers

**Figure 7:**
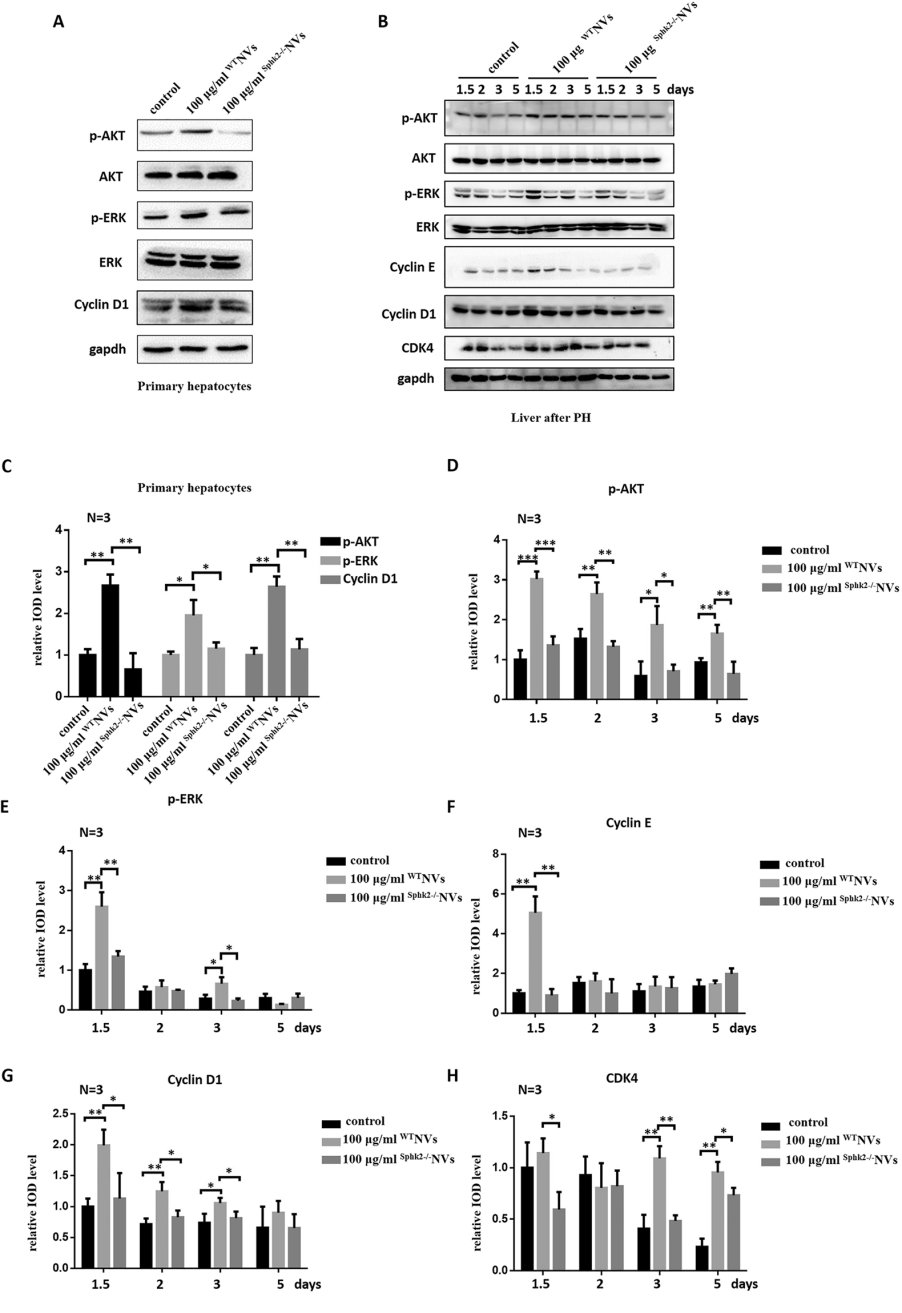
Exosome-mimetic NVs activated AKT and ERK phosphorylation in hepatocytes and livers. (**A**) Primary hepatocytes were treated with PBS (control), ^WT^NVs at a final concentration of 100 μg/ml or ^Sphk2−/−^NVs at a final concentration of 100 μg/ml for 48 h. Then, the levels of AKT, p-AKT, ERK, p-ERK and cyclin D1 in primary hepatocytes were evaluated via western blotting. (**B**) Immediately and every 24 h after a two-thirds PH was performed in mice, the mice were treated with PBS (control), 100 μg of ^WT^NVs or 100 μg of ^Sphk2−/−^NVs, and the levels of p-AKT, p-ERK, cyclin D1, cyclin E and CDK4 in the livers were evaluated via western blotting. (**C**–**H**) The relative IOD level was also analyzed. *P < 0.05; **P < 0.01; ***P < 0.001. Uncropped western blots are shown in Supplementary Figure 1.

AKT and ERK have long been known to play important roles in the regulation of cell proliferation. S1P has been confirmed to regulate the phosphorylation of ERK1/2 and AKT^36,37^. We measured the p-ERK1/2 and p-AKT levels in primary hepatocytes treated with ^WT^NVs, ^Sphk2−/−^NVs or PBS vehicle. The data showed that upregulation of SphK2/S1P by ^WT^NVs activated the phosphorylation of AKT and ERK1/2 (Fig. 7A). However, when primary hepatocytes were treated with ^Sphk2−/−^NVs, there was no obvious phosphorylation of AKT and ERK compared with hepatocytes treated with ^WT^NVs. To investigate whether exosome-mimetic NVs play an important role in regulating cell cycle, we measured the protein level of cyclin D1, which is a key cell cycle regulator. The results showed that treatment with ^WT^NVs increased the level of cyclin D1, while treatment with ^Sphk2−/−^NVs did not increase the level of cyclin D1 (Fig. 7A). We also measured p-ERK1/2 and p-AKT levels in livers from mice treated with ^WT^NVs, ^Sphk2−/−^NVs or PBS vehicle throughout the entire time-course after PH, and a similar phenomenon was observed (Fig. 7B). Regarding the protein level of cyclin D1, treatment with ^WT^NVs could slightly increase the level of cyclin D1, and treatment with ^Sphk2−/−^NVs had no effect (Fig. 7B). In addition, we found that after 36 h, the level of cyclin E protein in livers treated with these NVs was increased compared with that following treatment with PBS control or NVs from Sphk2−/− mice (Fig. 7B), which confirmed that these NVs play a role in the early stages of liver regeneration. We also found that 3 days after PH, the level of CDK4 protein was increased after treatment with these NVs compared with that following treatment with PBS control or NVs from Sphk2−/− mice (Fig. 7B). In addition, the intensity of protein bands was assessed using ImageJ software (Fig. 7C–H)

Taken together, these results suggest that increased formation of intracellular S1P by Sphk2 activated the phosphorylation of AKT and ERK and improved the expression of cyclin E, cyclin D1 and CDK4, which promoted hepatocyte proliferation and liver regeneration after PH.

## Discussion

In recent years, many studies on the involvement of exosomes in tissue repair and regeneration have been conducted because they can transport various biological molecules (protein, mRNA and miRNA) to recipient cells^33,38–40^. For example, exosomes from AKT gene-modified human umbilical cord mesenchymal stem cells could improve cardiac regeneration and promote angiogenesis by activating growth factor D derived from platelets^38^. Zhang *et al*. found that exosomes combined with β-TCP scaffolds could enhance osteogenesis by activating the PI3K/AKT signaling pathway, which may have an important role in repairing bone defects^39^. In addition, exosomes derived from Schwann cells could enhance axonal regeneration by mediating neuron-glia communication, and this finding has enormous significance in nerve repair and functional reconstruction^40^. Moreover, treatment with exosomes from primary hepatocytes could effectively increase hepatocyte proliferation and liver regeneration through upregulation of Sphk2^33^, and high levels of SphK2 have been confirmed to be associated with cancer growth and insensitivity to chemotherapy^41,42^. Although there have been many studies focusing on SphK2, the functions of SphK2 have remained a mystery^41^.

Although the potential of exosomes in regenerative medicine is widely understood, obtaining exosomes from cell cultures is very limited, and the purification process is very complicated. Development of new strategies for regenerative medicine is very important for clinic application. Exosome-mimetic NVs, the preparation technology of which has been established in recent years, could efficiently deliver siRNA and chemotherapeutics to recipient cells and have some characteristics similar to exosomes^28–30^. In addition, another report has shown that the use of exosome-mimetic NVs from pancreatic β-cells could efficiently induce differentiation of therapeutic insulin-producing cells from BM cells *in vivo*^31^. Based on these reports, we assume that exosome-mimetic NVs may have biological functions similar in some ways to exosomes. Here, we found, for the first time, that exosome-mimetic NVs from primary exosomes have a biological function in promoting cell proliferation and liver regeneration similar to that of exosomes from hepatocytes.

The present results showed that exosome-mimetic NVs derived from primary hepatocytes could promote recipient hepatocyte proliferation *in vitro* and liver regeneration *in vivo* by delivering Sphk2 to hepatocytes. Additionally, delivery of Sphk2 could increase the synthesis of intracellular S1P, which is a protective mechanism by which the liver recovers from such damage. Upregulation of Sphk2 and intracellular S1P would increase AKT and ERK phosphorylation. Based on the increased hepatocyte proliferation, we also measured cyclin E, cyclin D1 and CDK4 in livers after PH. The data showed that NVs from primary hepatocytes could improve the expression of cyclin E, cyclin D1 and CDK4. Furthermore, to test whether Sphk2 contained in exosome-mimetic NVs was the key factor in promoting liver regeneration, we used Sphk2-knockout NVs to confirm the hypothesis. We found that knockout of Sphk2 removed the effect of NVs on hepatocyte proliferation and liver regeneration. Finally, our findings that exosome-mimetic NVs and exosomes from primary hepatocytes both contain Sphk2, which promotes primary hepatocyte proliferation *in vitro* and liver regeneration *in vivo* support the hypothesis that exosome-mimetic NVs may be similar to exosomes in several respects, especially in bioactivities, which is relevant to clinical application.

Previous studies have found that exosome-mimetic NVs have a utilization potential similar to that of exosomes and that they could deliver exogenous molecules, such as siRNA and chemotherapeutics, to recipient cells^28–30^. However, no studies have indicated that exosome-mimetic NVs have biofunctions similar to those of exosomes. In this study, we prepared exosome-mimetic NVs from primary hepatocytes with characteristics similar to exosomes from primary hepatocytes, such as zeta potential and exosomal markers, that contained specific proteins that are also contained in exosomes from primary hepatocytes, such as Sphk2. We found that the NVs could efficiently deliver the specific endogenous molecule Sphk2 to recipient cells. Based on these experiments, the exosome-mimetic NVs could also promote hepatocyte proliferation and liver regeneration, similar to exosomes from primary hepatocytes. These results suggest that exosome-mimetic NVs from primary hepatocytes may serve as a new tool to promote the development of regenerative medicine. Strategies inspired by this study may lead to substitution of exosomes with exosome-mimetic NVs for biofunctional or clinical applications, including utilization in tissue repair and regeneration, as well as other applications.

Overall, our results illustrate the potential of substituting exosome-mimetic NVs for exosomes as cell-free therapeutics that can provide new strategies for regenerative medicine. The selection of appropriate sources of exosome-mimetic NVs and the optimization of extracellular matrix components will likely promote the development of regenerative medicine in the future. Exosome-mimetic NVs have several advantages compared to exosomes for clinical application, such as high yield and a simple purification processes during production. The results suggest that exosome-mimetic NVs could possibly be used as a new tool in tissue repair and regeneration.

## Materials and Methods

### Preparation of primary hepatocytes, exosomes and exosome-mimetic NVs

Primary hepatocytes were isolated from mice as previously described^42,43^. Hepatocytes were cultured in Dulbecco’s modified Eagle’s medium (DMEM; Gibco, Gaithersburg, MD) and supplemented with 10% fetal bovine serum (FBS) (Gibco, Gaithersburg, MD), 100 U/ml penicillin and 100 μg/ml streptomycin and maintained at 37 °C in an incubator with 5% CO2. Exosomes were prepared as previously described^14^. Hepatocytes were cultured in 10% exosome-free FBS for 48 h, and the supernatant was collected. Exosomes were isolated from the supernatant by differential centrifugation. Exosome-mimetic NVs were prepared according to the method established by Su Chul Jang with minor modification^29^. The adherent primary hepatocytes were detached by scraping and resuspended in phosphate-buffered saline (PBS). The cell suspension (5 × 10^6^ cells/ml) was serially extruded five times through 10-μm, 5-μm and 1-μm polycarbonate membrane filters (Nuclepore, Whatman, Inc., Clifton, NJ) using a mini-extruder (Avanti Polar Lipids, Birmingham, AL)^29,30^. To perform two-step OptiPrep density gradient ultracentrifugation, 50% iodixanol (1 ml, Axis-Shield PoC AS, Oslo, Norway), 10% iodixanol (2 ml) and the sample (7 ml) were placed in an ultracentrifuge tube from bottom to top and then ultracentrifuged at 100,000 g for 2 h at 4 °C. NVs were obtained from the interface of the 50% and 10% iodixanol layers and then washed with PBS 3 times. The NVs were further resuspended in PBS. The NVs were filtered through a 0.45-μm filter and stored at −80 °C until use. The protein content of the exosome-mimetic NVs was determined using a BCA protein assay kit (Thermo Scientific, USA).

### Mice and partial hepatectomy (PH)

Male C57Bl/6 mice were purchased from Model Animal Research Center of Nanjing University, and Sphk2-knockout mice were purchased from Jackson Laboratory (kindly provided by Dr. Richard L. Proia). All animals used for experiments were 6- to 8-week-old male mice. All the animal protocols were reviewed and approved by the Animal Care Committee of Nanjing University in accordance with the guidelines of the Institutional Animal Care and Use Committee. A two-thirds PH was performed with removal of the gallbladder between 8 and 12 am^44^. The mice were intravenously injected with exosome-mimetic NVs (unless otherwise indicated, “NVs” stands for NVs from wild-type mouse-derived primary hepatocytes) or PBS (vehicle control) immediately after and 24 h after PH. The mortality rate of the mice after PH was less than 5%. At the indicated time points, the mice were sacrificed to obtain liver fragments and serum.

### Transmission electron microscopy (TEM) and size and zeta potential measurements

Exosome-mimetic NVs were examined by TEM as previously described with minor modifications^14^. NVs (10 μg of total NV protein) were applied to copper-mesh Formvar grids (Beijing Zhong Xingkeyi Technology Co., Ltd., Beijing, China) and negatively stained with 2% phosphotungstic acid. TEM images were obtained using a JEM-2100 transmission electron microscope (Jeol, Japan). For size and zeta potential measurements, NVs were resuspended in PBS (5 μg of total NV protein), and the size and zeta potential of the NVs were measured with a Zetasizer Nano ZS90 instrument (Malvern, UK).

### Hepatocyte proliferation analysis

Hepatocyte proliferation was measured using MTT assays and DNA incorporation of 5-bromo-2-deoxyuridine (BrdU) *in vitro*. Hepatocytes were cultured on 96-well plates at a concentration of 5 × 10^3^ cells/well and treated with or without NVs for 24 or 48 h. For the MTT assays, MTT solution (5 mg/ml, 20 μl/well) was added to the 96-well plates. After incubation for 4 h, 150 μl of DMSO was added to dissolve the non-soluble crystals. The absorbance was measured by a spectrophotometer at 490 nm. For BrdU incorporation, hepatocyte proliferation was determined using a BrdU cell proliferation ELISA system (Abcam, Cambridge, UK) according to the manufacturer’s instructions. All experiments were performed with 3 replicates.

### Histological Analysis

Liver tissue was fixed in 4% paraformaldehyde for at least 24 h and then embedded in paraffin. Slices were subjected to anti-Ki67 immunohistochemical staining. To measure hepatocyte proliferation, six high-powered fields from every section were analyzed to obtain an average number of Ki67-positive cells.

### PKH67 labeling and assay to examine NV uptake by hepatocytes

NVs (20 μg) were labeled using a PKH67 Green Fluorescent Cell Linker Kit for General Cell Membrane Labelling (Sigma-Aldrich) as described previously^30^. PKH67-labeled NVs were added to hepatocytes and incubated for 12 h at 37 °C in 5% CO2. The cells were washed twice with PBS after incubation, fixed with 4% formaldehyde for 15 min, and washed twice with PBS again. The cells were counterstained with DAPI before being mounted with ProLong Gold Antifade Reagents. The uptake of NVs by hepatocytes was measured via fluorescence microscopy.

### Enzyme-linked immunosorbent assay (ELISA)

Quantification of sphingosine1-phosphate (S1P) in cell lysates by ELISA was performed using a Sphingosine-1-Phosphate Assay Kit (S1P-ELISA, DEIA-XYZ5, Creative Diagnostics, NY, USA) according to the manufacturer’s instructions, and optical density (OD) was measured at 450 nm.

### Western blotting

NVs and liver proteins were separated by SDS-PAGE (8–12% resolving gel) and transferred onto a polyvinylidene difluoride membrane, and the membrane was blotted with anti-CD9, anti-CD63 (System Biosciences), anti-Sphk2 (Abcam Inc.), anti-p-ERK (Cell Signaling Technology), anti-p-AKT (Cell Signaling Technology), anti-cyclin D1 (Cell Signaling Technology), anti-CDK4 (Cell Signaling Technology) and anti-cyclin E (Santa Cruz Biotechnology) and incubated with HRP-conjugated secondary antibody. Then, the membrane was incubated in ECL solution (Millipore, Switzerland) and imaged using a Tanon 5200 imaging system (Tanon, China). The intensity of protein bands was assessed using ImageJ software.

### ALT and AST detection

Serum ALT and AST levels, which are markers of liver function or injury, were determined using ALT and AST detection kits (Nanjing Jiancheng Bioengineering Institute, Nanjing, China) according to the manufacturer’s protocol.

### Statistical analysis

All results are expressed as the means ± standard deviation (SD). The data were analyzed with Students’ t-test. At P < 0.05, the data were considered significantly different.

The datasets generated during and/or analyzed during the current study are available from the corresponding author upon reasonable request.

## Electronic supplementary material

supplementary information

supplementary table S1
