# Supplementary material for: Exosome-Mimetic Nanovesicles from Hepatocytes promote hepatocyte proliferation in vitro and liver regeneration in vivo
Source: Sci Rep. 2018 Feb 6;8:2471. doi: 10.1038/s41598-018-20505-y (PMC5802835; doi:10.1038/s41598-018-20505-y)
Supplement: Supplementary file 1 — supplementary information [file 41598_2018_20505_MOESM1_ESM.pdf]

# **Exosome-Mimetic Nanovesicles from Hepatocytes promote hepatocyte proliferation in vitro and liver regeneration in vivo**

Jun-Yi Wu[1#], An-Lai Ji[2#], Zhong-xia Wang[1#]， Guang-Hui Qiang[3], Zhen Qu[1], , Jun-Hua Wu[4*], Chun-Ping Jiang[1*]

Supplementary Figure 1: Unprocessed scans of original western blots used in the main figures

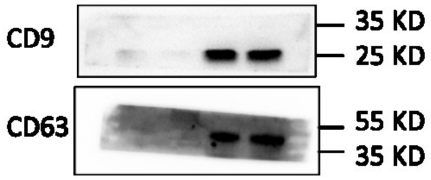


Blots for Figure 1D

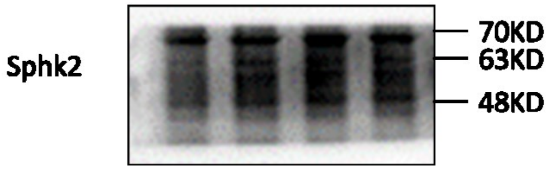


Blots for Figure 6B

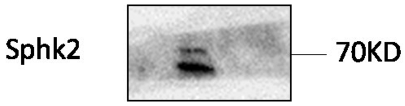


Blots for Figure 6D

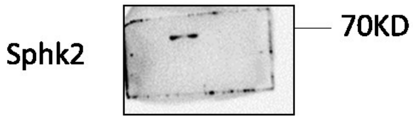


Blots for Figure 6E

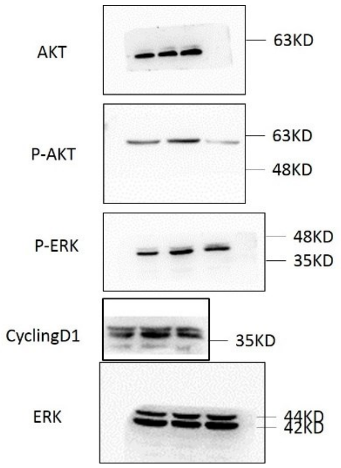


Blots for Figure 7A

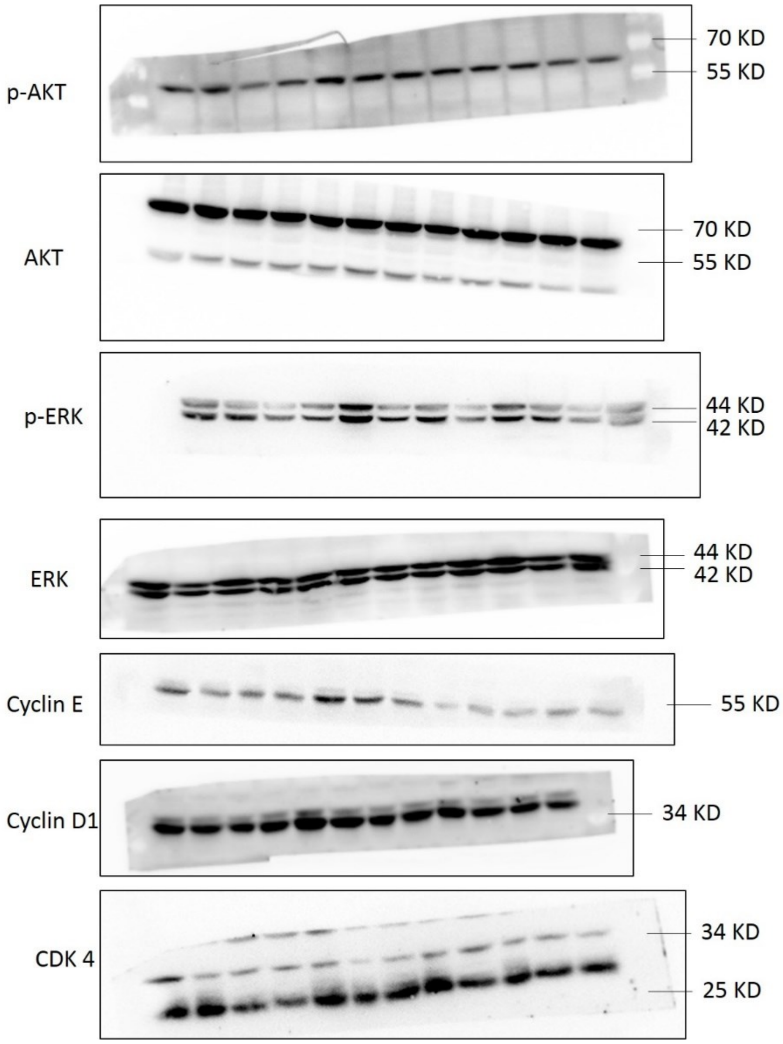


Blots for Figure 7B

Table S1. The proteins contained in both the NVs and exosomes were detected via mass spectrometry for the proteomic analysis. A total of 428 proteins contained in both the NVs and exosomes are listed.
